# Diagnostic potential of salivary biomarkers for primary biliary cholangitis: a systematic review

**DOI:** 10.3389/fmed.2025.1670206

**Published:** 2026-01-15

**Authors:** Mahnoor Saeed, Muhammad Saad Shaikh, Alhanouf Binhezaim, Tahani Almutairi, Mohid Abrar Lone, Syed Jawad Ali Bukhari, Muhammad Sohail Zafar

**Affiliations:** 1 Sindh Institute of Oral Health Sciences, Jinnah Sindh Medical University, Karachi, Pakistan; 2 Department of Oral Biology, Sindh Institute of Oral Health Sciences, Jinnah Sindh Medical University, Karachi, Pakistan; 3 Department of Pedodontics, Consultant Pediatric Dentist, Prince Sultan Military Medical City, Riyadh, Saudi Arabia; 4 Department of Oral Pathology, Sindh Institute of Oral Health Sciences, Jinnah Sindh Medical University, Karachi, Pakistan; 5 Estrabillo Dental Group, Ancaster, ON, Canada; 6 Department of Periodontology, Azra Naheed Dental College, Superior University, Lahore, Pakistan; 7 Clinical Sciences Department, College of Dentistry, Ajman University, Ajman, United Arab Emirates; 8 Centre of Medical and Bio-allied Health Sciences Research, Ajman University, Ajman, United Arab Emirates; 9 School of Dentistry, University of Jordan, Amman, Jordan

**Keywords:** salivary biomarker, primary biliary cholangitis, non-invasive diagnosis, anti-mitochondrial antibody, immunoglobulin A

## Abstract

**Background:**

Recent studies have explored saliva as a non-invasive diagnostic fluid across various systemic and autoimmune conditions. However, its potential role in diagnosing primary biliary cholangitis (PBC) remains unclear.

**Aim:**

This systematic review examines whether salivary biomarkers can assist in the diagnosis of PBC.

**Materials and methods:**

Indexed databases (PubMed and Scopus) and bibliographies of relevant articles were searched between November 2024 and December 2024. Original cross-sectional studies investigating salivary biomarkers specifically for PBC diagnosis were included. Quality appraisal was conducted using the Joanna Briggs Institute (JBI) critical appraisal checklist.

**Results:**

Three studies involving 204 participants met the inclusion criteria. Salivary antimitochondrial antibodies-M2 (AMA-M2), pyruvate dehydrogenase complex E2 (PDC-E2) related autoantibodies, and inflammatory cytokines [interleukin (IL)-6, IL-17A, interferon gamma (IFN-γ), tumour necrosis factor alpha (TNF-α)] were consistently elevated in PBC patients compared with healthy controls. Proteomic alterations, particularly increased cystatin S-type proteins and altered S100A family proteoforms, also differentiated PBC from healthy groups. Among all markers, AMA-M2 and PDC-E2 specific antibodies demonstrated the highest diagnostic specificity.

**Conclusion:**

Available evidence suggests that select salivary autoantibodies and inflammatory proteins may hold diagnostic potential for PBC. Although promising, current data are limited, and larger, standardised studies are required to validate these biomarkers for clinical use.

## 1 Introduction

Primary biliary cholangitis (PBC), formerly termed primary biliary cirrhosis up to 2016 (1), is a prototypic chronic autoimmune liver disease, characterised by damage to cells present in the bile duct. Destruction of these cells leads to the impairment of the intrahepatic bile duct (2). This immune response initiates when malfunctioning biliary epithelial cells (BECs) act as antigen-presenting cells (APCs), activating T-lymphocytes. Activated T cells attack the ubiquinone pyruvate dehydrogenase complex E2 (PDC-E2) molecules, followed by the formation of apoptotic blebs that progress into inflammation of the bile duct, a hallmark of a condition referred to as PBC (1).

Multiparous women are more susceptible to PBC due to several reasons, including elevated levels of oestrogen during pregnancy, which decrease the fluidity of the bile duct’s cell membrane, eventually blocking the absorption and excretion of bile acids (BAs) (3). Foetal microchimerism may also be involved in the etiopathogenesis of the disease (3). Moreover, this autoimmune disease can be genetically inherited or environmentally triggered (4). The human leukocyte antigen (HLA) gene significantly influences the probability of PBC. Moreover, its genetic predisposition is evident in a study highlighting a 63% similarity index in monozygotic twins (5) and a study held in Iceland that demonstrated the familial risk of PBC in the first-degree relative, primarily extending to second, third, fourth, and fifth-degree relatives (6). As environmental triggers, PBC could be associated with urinary tract infections, smoking, xenobiotic-induced modification, and, most importantly, bacterial infections (7).

Saliva is a colourless, odourless hypotonic solution with a pH of 6.6–7.1. The fluid shows a greater ability to serve as a health indicator and functions as an essential component of the nonspecific immune system (8). Considering its worth as a health indicator, saliva was first used for the differential diagnosis of Cushing’s syndrome (9).

Salivary fluid is a biological secretion. It contains 99% of water and 1% of mineral salts, along with organic compounds which comprise cholesterol, uric acid, and, proteins. The proteins in the saliva include immunoglobulins, lactoferrin, peroxidase, glycoprotein, and cytokines (10). Additionally, saliva contains more than 700 microbiomes that can be utilised for pathogenesis and diagnosis of diseases (8). Salivary biomarkers are measurable molecules in saliva; like proteins, antibodies, or cytokines that can indicate health or disease status. Supported by emerging evidence, a connecting link between blood and saliva can be hypothesised, whereby transcellularly or paracellularly serum molecules enter the salivary tissues altering the composition of saliva and thus helping in an individual’s health assessment (11).

Being non-invasive, safe to collect, easy to handle, store, and process, saliva is now considered a more useful, valuable, and preferred choice of diagnosis as compared to serum (12). Prior studies witness the application of saliva in diagnostic analysis of several diseases, such as caries (13), oral cancer (14), periodontal diseases (14), diabetes mellitus (15), Down syndrome (16), and denture-induced stomatitis (14).

Secretions from saliva and other ductal organs share similarities in terms of antigens and tissue features; hence, salivary dysfunction is noticeable in many self-reactive immune disorders such as immune-mediated hepatitis (17), primary sclerosing cholangitis (PSC) (18), lupus erythematosus disease (19), rheumatoid arthritis (20) and autoimmune thyroid diseases (21). Besides oral lesions being common in most autoimmune disorders (22), xerostomia is one of the symptoms of PBC, reported in patients due to its association with Sjogren’s syndrome (23).

Considering the interconnection of saliva with several diseases, particularly autoimmune diseases, and the diagnostic challenges faced in resource-challenged settings as addressed by a study conducted in Nigeria (24), we hypothesise that saliva might be an important diagnostic tool for diagnosing PBC, which is a rare autoimmune disorder with a point prevalence rate of 22.27 cases per 100,000 inhabitants (25). Therefore, this systematic review aims to analyse whether or not salivary biomarkers can help in the diagnosis of PBC.

## 2 Methods

This review adhered to the updated Preferred Reporting Items for Systematic Reviews and Meta-Analysis (PRISMA) guidelines (26).

### 2.1 Research question

This study was planned to answer the research question, “Can salivary biomarkers play a role in the diagnosis of primary biliary cholangitis (PBC)?”

### 2.2 Literature search

Different electronic databases (PubMed and Scopus) were extensively searched for the identification of the most relevant literature on assessing the significance of salivary biomarkers in the diagnosis of PBC. The literature search was conducted between November 2024 and December 2024. The keywords searched for the most relevant studies were “primary biliary cholangitis” OR “primary biliary cirrhosis” AND “saliva” OR “salivary biomarkers” OR “non-invasive diagnosis.” For more refined results, literature from a manual search was also obtained. Details related to the search strategy can be seen in the Supplementary file.

We used free-text keywords combined with Boolean operators to maximize retrieval across databases. MeSH terms were not applied because relevant studies lacked consistent MeSH indexing, making free-text searching more appropriate for this emerging topic.

### 2.3 Literature screening

All retrieved citations were imported into EndNote 20, where both automatic and manual deduplication procedures were performed to remove internal and external duplicates. The deduplicated dataset was then exported into Microsoft Excel for title/abstract screening and full-text assessment. Two authors (MS and MSS) independently screened all records, and any disagreements were resolved through discussion with a third author (MSZ).

### 2.4 Eligibility criteria

This review follows the following criteria for selection of studies:

#### 2.4.1 Inclusion criteria

This review includes original studies such as descriptive and analytical cross-sectional studies, cohort studies, case–control studies, and randomised controlled trials (RCTs) for investigating biomarkers that play a pivotal role in the diagnosis of PBC. Moreover, studies that exclusively used saliva for diagnostic purposes were included. This focus was chosen to minimize heterogeneity and to emphasize the translate oral diagnostic potential of salivary biomarkers in PBC Participants, irrespective of demographics, were included in the review. This study includes only articles that are published in the English language and conducted on humans.

#### 2.4.2 Exclusion criteria

Review articles, case reports, unpublished data, short communications, letters to the editor, book chapters, grey literature and non-indexed studies were excluded from the study. Studies that provided only the difference between the salivary biomarker profile of healthy controls and PBC patients and did not mention their use for diagnostic applications were excluded from the study. Biomarkers investigated in diseases other than PBC were excluded from the study. This systematic review excludes all the research papers that presented relevant information in languages other than English.

### 2.5 Data extraction

Included studies were read in detail, and information such as the name of the author, study design, biomarkers in saliva investigated, patient population, diagnostic techniques used, and the results were extracted from each study. For the data extraction process, two authors (MS and MSS) independently extracted data from the included studies. Any conflict in opinion was settled by agreement with the third author (MSZ).

### 2.6 Risk of Bias

To assess the risk of bias in each included research paper, the updated version of the Joanna Briggs Institute (JBI) Critical Appraisal Checklist for analytical cross-sectional studies (27) was used, which is specialised for focusing on the sample’s selection criteria, methods of collecting data, statistical analysis and assessing the validity of outcomes. Moreover, the tool presents a percentile to estimate the quality of included studies. Quality ranking was allocated as low (less than 49%), medium (50–69%), or high (above 70%) (28). For the reviewing process, two authors (MS and MSS) independently reviewed each study thoroughly.

## 3 Results

### 3.1 Screening results

**Figure 1:**
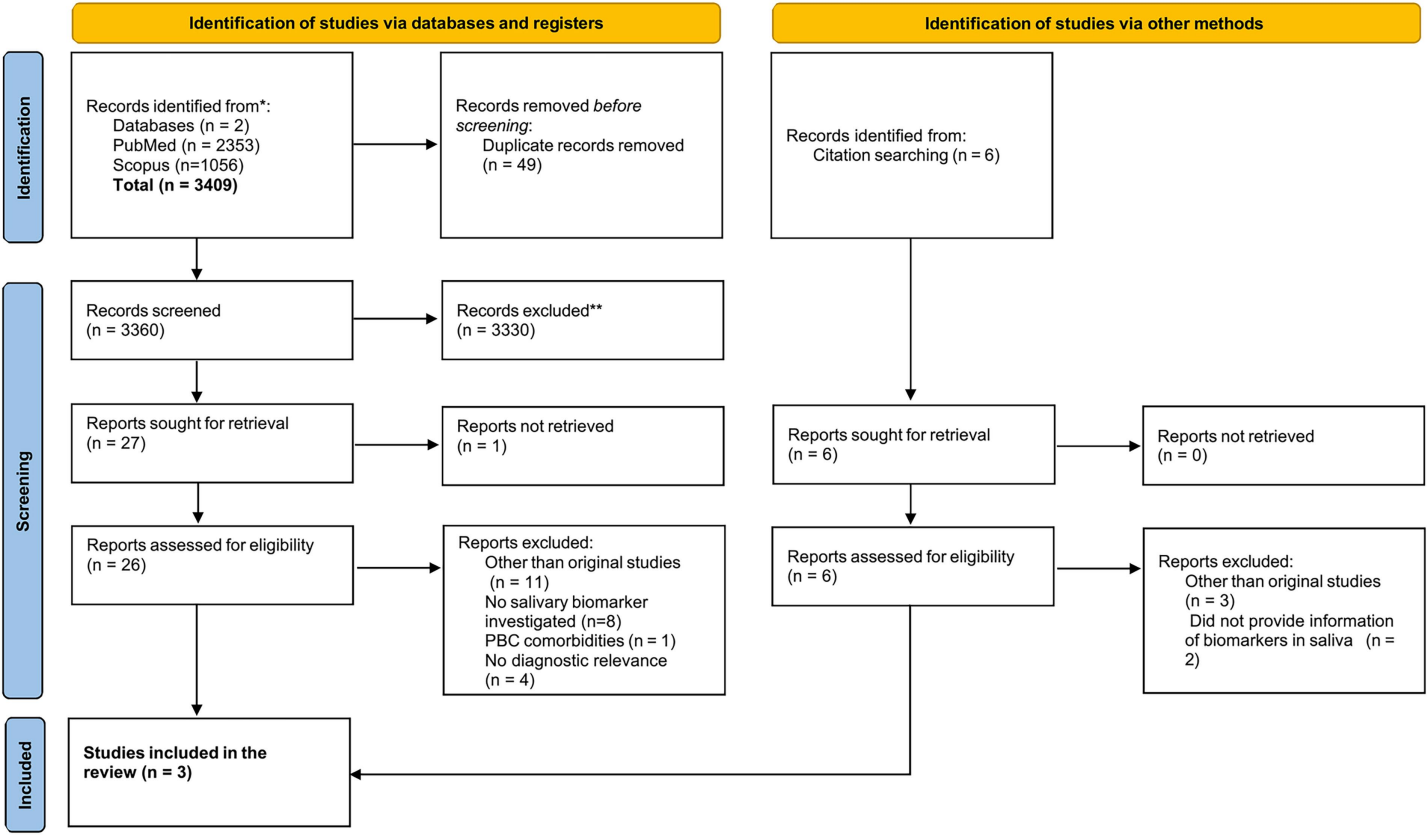
PRISMA flowchart for selection process of the publications included.

An electronic search of PubMed and Scopus was performed, identifying 2,353 and 1,056 records, respectively. After removing all internal and external duplicates, the results shrank to 3,360. Title and abstract screening removed all the irrelevant studies and narrowed the results to 27 articles. The full-length publication of 26 articles was accessed and read thoroughly. However, we could not retrieve the full text of one article. Apart from electronic databases, a manual search was also made. Citation searching presented a total of six articles. After screening, five studies were excluded for the reasons mentioned below, while one study was added to our review. After a careful evaluation, three articles were included in the study for critical appraisal. The flowchart (Figure 1) provides a summary of all the results obtained (26). The reason for the exclusion of 26 studies has been given in Table 1.

**Table 1:** Reason for exclusion of studies after full-text evaluation.

Author/year	Reference	Reason for exclusion
Bogdanos et al. (2003), Hirschfield et al. (2011), Long et al. (2002), Younossi et al. (2019), Bizzaro et al. (2012), Bogdanos et al. (2011), Floreani et al. (2002), Gao et al. (2015), Hu et al. (2010), Jones et al. (2000), Selmi et al. (2008), Berg et al. (1997), Berg (1986), Tsuneyama et al. (1995).	(41–54)	Not original studies.
Floreani et al. (2002), Chalifoux et al. (2017), Copaci et al. (2002), Kikuchi et al. (2005), Fukushima et al. (2002), Corpechot et al. (2022), Gabeta et al. (2008), Morshed et al. (1992), Olmez et al. (2016), Joplin et al. (1994), Bowlus et al. (2014).	(47, 55–64)	These did not choose saliva samples or did not include information about salivary biomarkers.
Tanaka et al. (2007).	(37)	PBC patients with other associated diseases.
Abe et al. (2018), Lv et al. (2021), Palmer et al. (2000), Reynoso et al. (2000).	(33–35, 65)	These did not mention whether the discussed biomarkers can be used for diagnostic purposes or not.

PBC, primary biliary cholangitis.

### 3.2 Type of study design

This systematic review comprises all the studies that follow a cross-sectional study design for the investigation of salivary biomarkers (29–31). Out of all studies published between 2001 and 2023, to our knowledge, only three studies fall into the inclusion criteria to answer our research question.

### 3.3 Characteristics of included studies

For the diagnostic purpose of the concerned disease (i.e., PBC), the varying levels of salivary proteomes and antibodies were investigated using different diagnostic techniques. Excluding patients with diseases other than PBC, 204 individuals were selected as a sample population. Participants in the included studies were categorised into two groups: PBC patients and the healthy group. Table 2 shows the general characteristics of the included studies.

**Table 2:** General characteristics of the included studies.

Author/year	Study design	Biomarkers in saliva investigated	Patients (*n*) male/female (M/F) and study groups	Diagnostic technique(s) used	Main results
Olianas et al. (2023) (29)	Analytical cross-sectional study	S100A12 S100A9 short cystatin S1, S2, SN & C	Patients (72) M/F: 08/64 (Only PBC and HCs group gender distribution mentioned here) Study groups: PBC: 36 HCs: 36 AIH: 36	Mass spectrometry	Cystatin S-type was higher in PBC patients than HCs. S100A12, S100A9 short, and aPRPs were lower in PBC than in HCs.
Lu et al. (2017) (30)	Analytical cross-sectional study	AMA-M2 IL-6 IL-17A IFN-γ TNF-α	Patients (109) M/F: 19/90 Study groups: PBC: 49 HCs: 60	ELISA	PBC patients with positive serum AMA-M2 showed positive saliva AMA-M2 levels. On the other hand, all participants from HCs showed negative results. IL-6, IL-17A, IFN-γ, and TNF-α showed increasing levels in the saliva of the PBC group compared to HCs.
Ikonu et al. (2001) (31)	Analytical cross-sectional study	AMA PDC-E2 enzyme, OGDC-E2 enzyme, BCOADC-E2 enzyme, IgG, IgA, IgM	Patients (23) M/F: 01/11 (Only PBC group gender distribution reported) Study groups: PBC: 12 HCs: 11	Immunoblotting Enzyme Inhibition Assay and Immunofluorescence	Out of 12 PBC patients, among 2-OADC enzymes; 75% of the PBC patients had antibodies against PDC-E2, 42% had antibodies against OGDC-E2 and 25% had antibodies against BCOADC-E2. The saliva of PBC patients showed higher intensity of IgG in all patients, IgA in four out of nine, and IgM in one out of nine patients compared to healthy group.

2-OADC, 2-oxoacid dehydrogenase complex; AIH, autoimmune hepatitis; AMA, anti-mitochondrial antibody; aPRPs, acidic proline-rich proteins; BCOADC-E2, branched chain 2-oxoacid dehydrogenase complex E2; ELISA, enzyme-linked immunosorbent assay; HCs, healthy controls; IFN-γ, interferon gamma; IgA, immunoglobulin A; IgG, immunoglobulin G; IgM, immunoglobulin M; IL, interleukin; OGDC-E2, oxoglutarate dehydrogenase complex E2; PBC, primary biliary cholangitis; PDC-E2, pyruvate dehydrogenase complex E2; TNF-α, tumour necrosis factor alpha.

### 3.4 Participants in the study

Out of 204 participants, 97 participating members were PBC patients, while 107 participants belonged to the healthy group. Among 97 PBC patients, 36 patients were contributors to research executed by Olianas et al. (29), 49 patients belonged to the study conducted by Lu et al. (30) and 12 patients belonged to the study conducted by Ikuno et al. (31).

### 3.5 Diagnostic techniques used

For the screening of salivary proteomes as biomarkers, a survey conducted by Olianas et al. used the Mass Spectrometry (MS) method as a diagnostic technique (29). Furthermore, Lu et al. used enzyme-linked immunosorbent assay (ELISA) to detect levels of antimitochondrial antibodies-M2 (AMA-M2) and cytokines (30). The study carried out by Ikuno et al. utilised immunoblotting, enzyme inhibition assay and immunofluorescence as diagnostic techniques (31).

### 3.6 Biomarkers investigated in the saliva

Olianas et al. investigated proteomes and peptides present in saliva, i.e., S100A family and cystatins, more precisely S100A12, S100A9 short, and cystatin S1, S2, SN, and C, respectively (29), while the analytical research authored by Lu et al. examined AMA-M2 and inflammatory cytokines such as Interleukin (IL)-6, IL-17A, interferon gamma (IFN-γ) and tumour necrosis factor alpha (TNF-α) as salivary biomarkers (30). Lastly, the biomarkers investigated in a study by Ikonu et al. include PDC-E2, oxoglutarate dehydrogenase complex E2 (OGDC-E2), branched chain 2-oxoacid dehydrogenase complex E2 (BCOADC-E2), and immunoglobulins (IgG, IgA, and IgM) (31).

### 3.7 Main results

After the analysis, Olianas et al. evaluated that cystatin S-type (cystatin S1, S2, and SN) and the sum of their related proteomes were increased in PBC patients compared to healthy individuals; however, the level of S100A9 proteoforms and aPRPs (PRP3_1P) were decreased (29). Lu et al. presented the result that salivary AMA-M2 is detectable only in PBC patients with positive serum AMA-M2 and inflammatory cytokines (IL-6, IL-17A, IFN-γ, and TNF-α) levels are elevated in diseased patients in comparison to the healthy group (30). Research by Ikonu et al. examined saliva samples of 12 PBC patients; 9 of them had antibodies against the PDC-E2 enzyme that inhibited their activity in saliva, 3 patients were reactive to BCOADC-E2 enzyme, and 5 were reactive to OGDC-E2 enzyme. Moreover, among 12 PBC patients, intensities of IgG, IgA, and IgM were elevated in comparison to healthy participants. However, the most predominant was IgG (31).

### 3.8 Quality appraisal

The quality of each analytical cross-sectional study was assessed to check the risk of bias using the set of questions provided by the JBI tool. All the included studies showed a high percentage of yes; the quality score ranged from 75 to 87.5% (high quality). The negative answers in the first and second studies came from Question # 6, which did mention the confounding factors but not the strategies to overcome these factors, and in the third study, Question # 2 did not explicitly mention the study subjects and study setting (location, demographics, and time). Question # 1 of the second study was unclear, as the study did not clearly define the selection criteria for the recruitment of the sample population. Table 3 demonstrates quality appraisal of the included studies utilising JBI critical appraisal checklist for cross-sectional studies.

**Table 3:** Quality appraisal of the included studies using JBI critical appraisal checklist.

Study/Domain	Q1	Q2	Q3	Q4	Q5	Q6	Q7	Q8	Overall appraisal
Olianas et al. (2023) (29)	Yes	Yes	Yes	Yes	Yes	No	Yes	Yes	87.5%
Lu et al. (2017) (30)	Unclear	Yes	Yes	Yes	Yes	No	Yes	Yes	75%
Ikuno et al. (2001) (31)	Yes	No	Yes	Yes	Yes	Yes	Yes	Yes	87.5%

^Q1^Were the criteria for inclusion in the sample clearly defined? ^Q2^Were the study subjects and the setting described in detail? ^Q3^Was the exposure measured in a valid and reliable way? ^Q4^Were objective, standard criteria used for measurement of the condition? ^Q5^Were confounding factors identified? ^Q6^Were strategies to deal with confounding factors stated? ^Q7^Were the outcomes measured in a valid and reliable way? ^Q8^Was appropriate statistical analysis used?

## 4 Discussion

The findings from the reviewed studies show varying levels of proteins, peptides, and antibodies in the salivary fluid of PBC patients that shall be investigated for the detection of the disease. All the studies included were high quality, with a percentage of yes ranging from 75 to 85.7%.

Tan et al. in their study enlightened the finding that serologic examination of antibodies, assessment of ultrasound, and magnetic resonance cholangiopancreatography imaging are recommended for diagnosis of PBC before proceeding towards liver biopsy (32), but did not address saliva as a non-invasive diagnostic source. Other studies presented a significant association of other salivary biomarkers with PBC but did not specify the contribution of these components towards clinical confirmation (33–36).

To find a role for diagnostic means, varying levels of (i) AMA-M2 and inflammatory cytokines and (ii) salivary proteomes and peptides are discussed below;

### 4.1 Role of antimitochondrial antibodies and inflammatory cytokines as salivary biomarkers

Biliary and salivary ductular epithelium share functional and structural similarities with each other; hence, among 2-OAD enzymes, PDC-E2-like materials, which are notably found in the cells of bile ducts, autoantibodies to PDC-E2 have also been noticed in salivary duct cells in the case of PBC, suggesting similar diseases affect both tissues. Antibodies are naturally present in the saliva of healthy individuals; however, specific AMA-M2, IgA isotype, and predominantly IgG increase in the diseased condition. These antibodies are originally formed in mucosal tissues, then travel through the serum into the bile or saliva via transcytosis, causing an inflammatory response and damaging the epithelial cells, and may lead to secondary Sjogren’s syndrome. On the other hand, IgM isotype is less frequent in saliva and shows weak efficacy in binding to the secretory component (30, 31). In addition to AMA-M2, certain inflammatory cytokines, such as IL-6 and 17A, IFN-γ, and TNF-α are notably elevated in the saliva of diseased participants in comparison to the healthy control, indicating another non-invasive diagnostic biomarker (30).

These findings align with the outcomes obtained from a survey by Tanaka et al., who illustrated the positive association of salivary AMA with 12 out of 26 PBC patients and IgA-anti-PDC-E2 with 6 out of 26 PBC patients, but none of the participants from healthy controls had any positive association (37).

A study conducted by Reynoso-Paz et al. slightly contrasts our findings that no significant difference in IgG and IgA is evident in the saliva of PBC patients. Still, IgM has a *p*-value less than 0.0001, demonstrating a close association of IgM with PBC patients’ saliva. Moreover, more than half of the PBC subjects had IgA antibody specificity against the PDC-E2 molecule but lacked the detection of BCOADC and OGDC-E2, maybe because their synthesising rate is lower than that of PDC-E2 (35).

### 4.2 Role of proteomes and peptides as salivary biomarkers

A study by Olianas et al. presented the most distinguishing proteomes that show differentiating levels in PBC vs. healthy individuals. These include S100A12, S100A9 short, cystatin S1, S2, SN, and C.

To the best of our knowledge, existing literature has not directly correlated the varying levels of the salivary S100A protein family with PBC but a study by Ma et al. explored that S100A12 is responsible for the migration and activation of macrophages, causing inflammation in biliary epithelial cells. Hence, the expression of serum S100A12 is enhanced in PBC patients (38).

The other distinguishing proteome is the cystatin family. Cystatins can be classified into three main types, of which Type 2 cystatins need to be under consideration. Cystatin C is an inhibitor of cathepsin-S (CTSS-S). CTSS-S plays a significant role in regulating various aspects of natural killer T cells activation which triggers inflammation, and contributes to immunological disorder. In autoimmune conditions, a physiological defence mechanism involves an increase in the levels of cystatin C which inhibits CTSS-S. This increased level of cystatin C not only highlights its role as a protective regulator but also supports its potential as a diagnostic biomarker for autoimmune diseases, particularly those involving BECs, such as PBC. Additionally, inhibition of CTSS-S inhibits MHC-II presentation and inflammation acting as a protective mechanism (29). In support of this finding, a study by Thanei et al. demonstrated the consequences of CTSS-S inhibition in autoimmune-triggered patients. The results revealed suppressed MHC class II presentation and reduced inflammation (39).

S-type cystatins, another subtype of Type 2 cystatins, comprise Cystatin Type S, SN, and SA. Cystatin S plays a pivotal role in regulating the mineral balance of the tooth, hence showing a connecting link with the oral environment. Additionally, cystatin SN and SA are inhibitors of cathepsin B, H, and L and show therapeutic use in response to liver injury (29).

Since Olianas et al. were the first to study cystatins and S100A protein profiles, Guadalupi et al. presented a comparison between the salivary proteome profiles of PBC-affected individuals vs. healthy controls. This demonstrated the increasing levels of Cofilin-1, Gelsolin, and Clusterin in affected individuals compared to the healthy group (36).

These findings suggest their saliva being non-invasive approach, can be used as first-line screening tool in resource-limited settings. Additionally, it can be used in clinical practice for tracking treatment response in affected individuals as biopsies and blood test cannot be repeatedly performed.

When comparing the different biomarker classes investigated in the three included studies, salivary autoantibodies, particularly AMA-M2 and immunoglobulins directed against components of the 2-oxoacid dehydrogenase complex (e.g., PDC-E2), emerge as the most PBC-specific candidates. These markers recapitulate the established serum autoantibody signature of PBC and were detected exclusively in patients with PBC, but not in healthy controls, suggesting high diagnostic specificity in saliva as well. In contrast, elevated inflammatory cytokines and altered salivary proteomes (e.g., S100A12, S100A9 short, cystatin S-type and cystatin C) likely reflect broader autoimmune and inflammatory pathways and may therefore be more useful as adjunctive markers within multimarker panels, or for disease stratification and monitoring, rather than as stand-alone diagnostic tools at this stage.

### 4.3 Limitations of the review

The investigations addressed above, in the review, may not be representative of the population due to the limited number of studies taken into account, despite conducting a thorough literature search, underscoring that salivary biomarker research in PBC is still at an early exploratory stage. Also, the scarcity of data on the disease limits the investigations to variability in salivary proteomes and antibodies only; This highlights the need for larger, well-designed studies to strengthen the evidence base. Also, some of the studies mentioned the comparative analysis of salivary biomarker profiles for PBC with other liver diseases, we excluded this comparison; this may introduce bias in the findings, considering only a selective view presented in the review. Moreover, the heterogeneity in the findings with respect to the biomarker(s) evaluated, outcome measures, scarcity of available data and diagnostic techniques used in the included studies did not allow us to do a meta-analysis.

Lastly, the diagnostic utility of salivary biomarkers in PBC must be interpreted with caution due to several potential confounders. Oral health conditions (periodontitis, caries, salivary gland dysfunction), overlap with Sjögren’s syndrome, medication use, variations in salivary flow rate, lifestyle factors, and pre-analytical inconsistencies may all influence salivary cytokines, proteomic signatures, and autoantibody levels.

### 4.4 Future research work

As evident by the availability of three studies, PBC can be considered to be diagnosed through a non-invasive approach. However, further well-conducted and comprehensive research work is still required to fill the gap and bring more insights. Furthermore, individual biomarkers in this field need to be studied to validate these findings. Also, future studies should incorporate strict oral-health screening, standardised saliva-collection protocols, and careful control for autoimmune comorbidities to more accurately define the specificity and clinical usefulness of these markers.

Interestingly, a study by Ceccherini et al. (40) identified cytoskeleton-remodelling proteins in the saliva of patients with autoimmune cholangiopathies, including PBC, the study primarily focused on PSC and did not evaluate these proteins as diagnostic markers for PBC. Therefore, while excluded based on our criteria, its findings highlight emerging proteomic pathways that may inform future PBC-focused salivary biomarker research.

## 5 Conclusion

Findings about the salivary proteomes (more specifically, cystatins and S100A protein families), inflammatory cytokines, and autoantibodies reported significantly altered levels in the diseased patients compared to the healthy group, suggesting that saliva may have potential for diagnosing PBC. Nevertheless, further studies need to be done to validate the findings.

## Data Availability

Publicly available datasets were analyzed in this study. This data can be found at: PubMed and Scopus.
